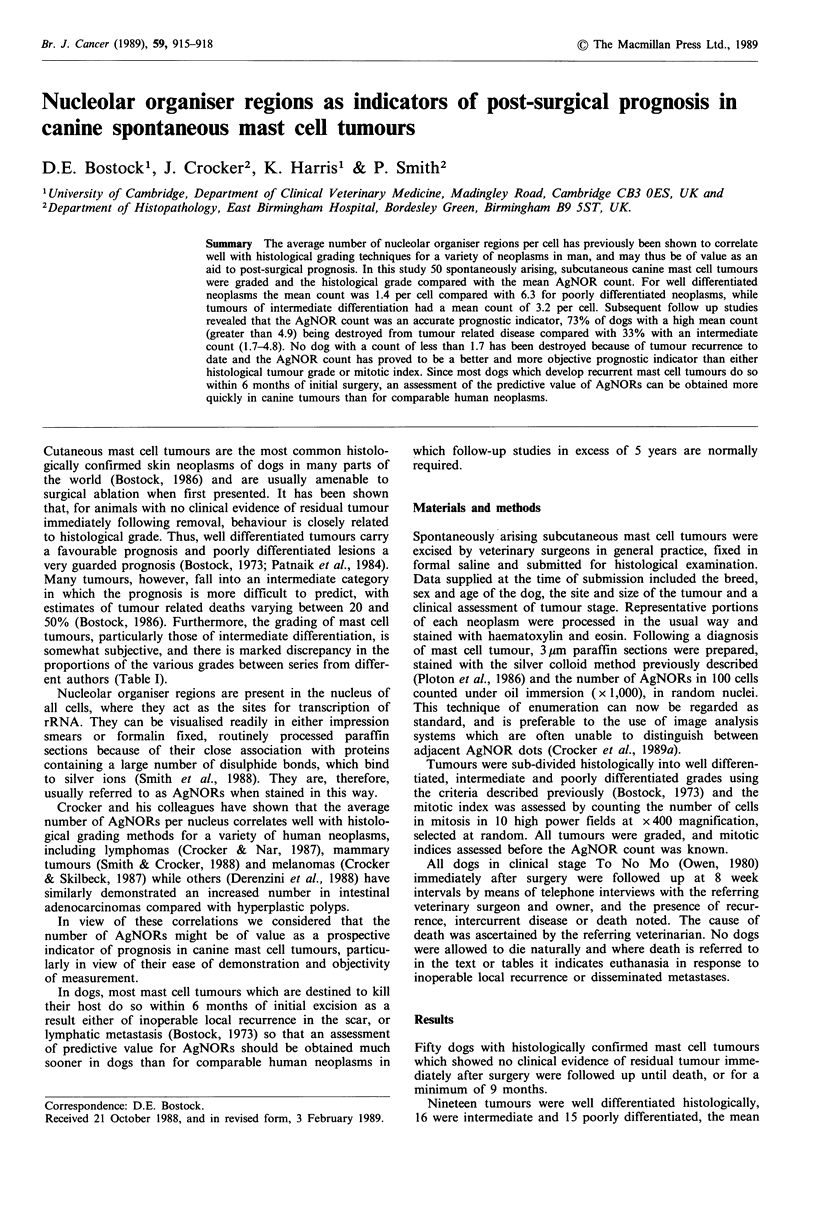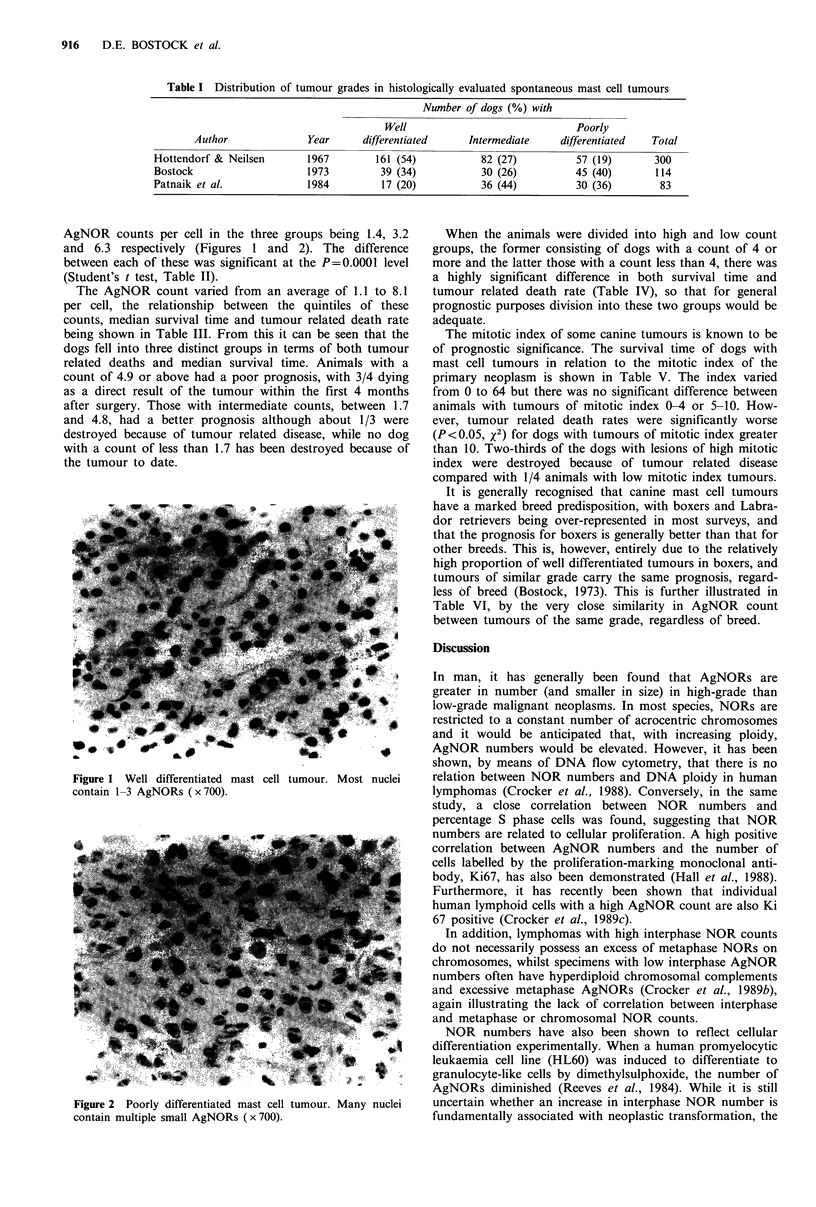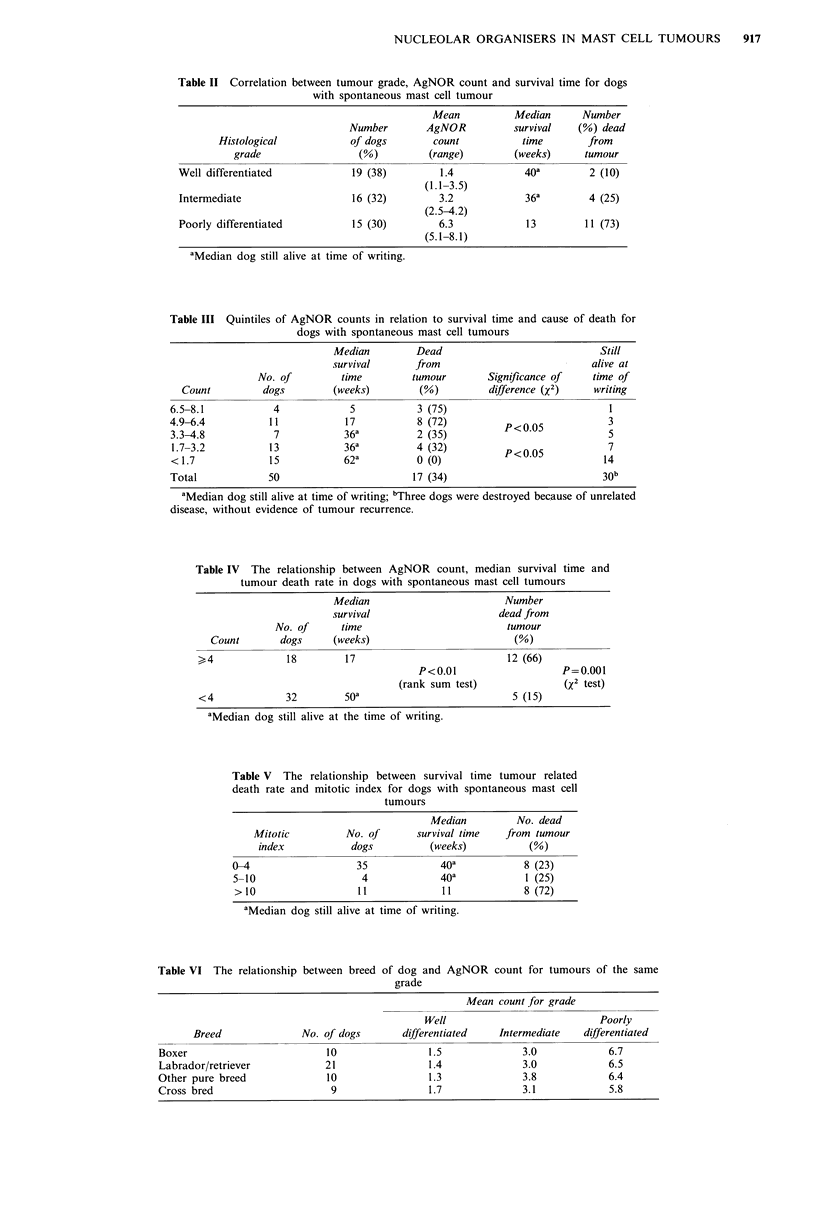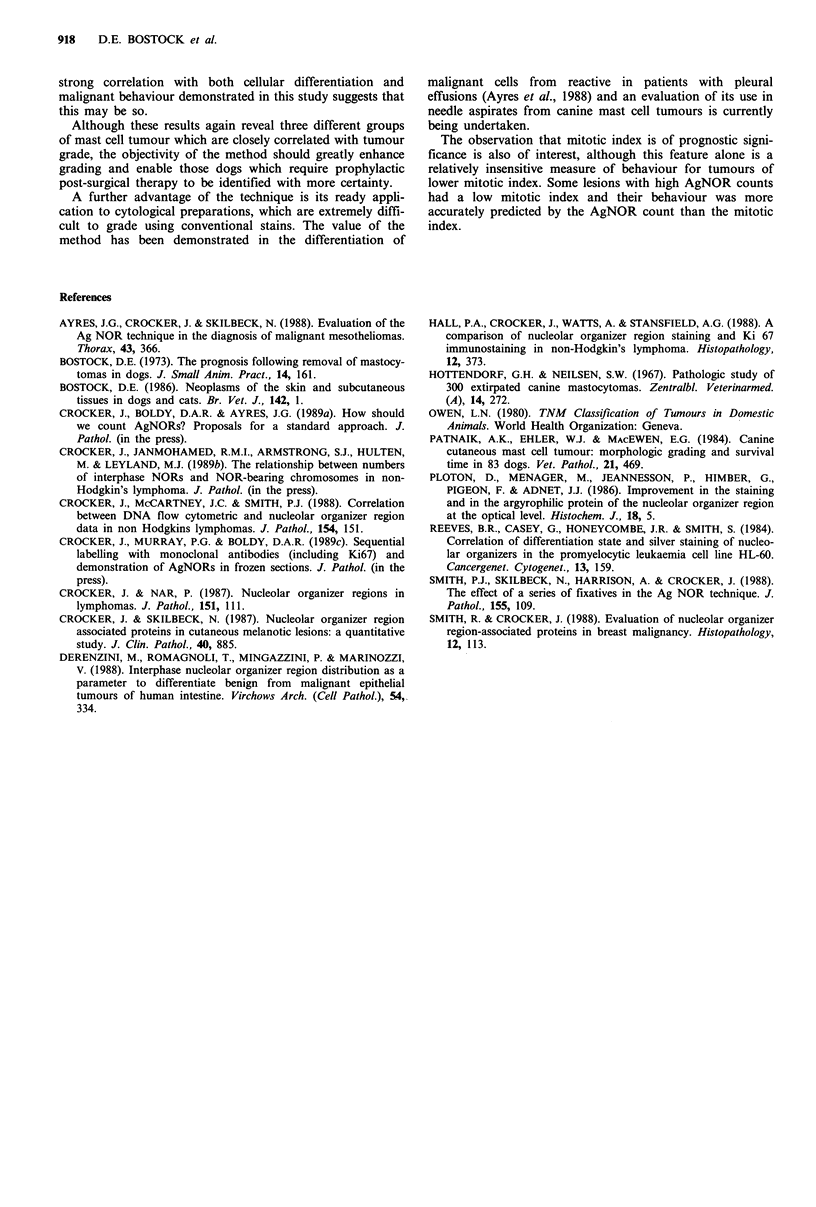# Nucleolar organiser regions as indicators of post-surgical prognosis in canine spontaneous mast cell tumours.

**DOI:** 10.1038/bjc.1989.193

**Published:** 1989-06

**Authors:** D. E. Bostock, J. Crocker, K. Harris, P. Smith

**Affiliations:** University of Cambridge, Department of Clinical Veterinary Medicine, UK.

## Abstract

**Images:**


*Br. J. Cancer* (1989), **59**, 915–918 © The Macmillan Press Ltd., 1989

### Nucleolar organiser regions as indicators of post-surgical prognosis in canine spontaneous mast cell tumours

D.E. Bostock[1], J. Crocker[2], K. Harris[1] & P. Smith[2]

[1]*University of Cambridge, Department of Clinical Veterinary Medicine, Madingley Road, Cambridge CB3 0ES, UK and* [2]*Department of Histopathology, East Birmingham Hospital, Bordesley Green, Birmingham B9 5ST, UK.*

**Summary** The average number of nucleolar organiser regions per cell has previously been shown to correlate well with histological grading techniques for a variety of neoplasms in man, and may thus be of value as an aid to post-surgical prognosis. In this study 50 spontaneously arising, subcutaneous canine mast cell tumours were graded and the histological grade compared with the mean AgNOR count. For well differentiated neoplasms the mean count was 1.4 per cell compared with 6.3 for poorly differentiated neoplasms, while tumours of intermediate differentiation had a mean count of 3.2 per cell. Subsequent follow up studies revealed that the AgNOR count was an accurate prognostic indicator, 73% of dogs with a high mean count (greater than 4.9) being destroyed from tumour related disease compared with 33% with an intermediate count (1.7–4.8). No dog with a count of less than 1.7 has been destroyed because of tumour recurrence to date and the AgNOR count has proved to be a better and more objective prognostic indicator than either histological tumour grade or mitotic index. Since most dogs which develop recurrent mast cell tumours do so within 6 months of initial surgery, an assessment of the predictive value of AgNORs can be obtained more quickly in canine tumours than for comparable human neoplasms.

Cutaneous mast cell tumours are the most common histologically confirmed skin neoplasms of dogs in many parts of the world (Bostock, 1986) and are usually amenable to surgical ablation when first presented. It has been shown that, for animals with no clinical evidence of residual tumour immediately following removal, behaviour is closely related to histological grade. Thus, well differentiated tumours carry a favourable prognosis and poorly differentiated lesions a very guarded prognosis (Bostock, 1973; Patnaik *et al.*, 1984). Many tumours, however, fall into an intermediate category in which the prognosis is more difficult to predict, with estimates of tumour related deaths varying between 20 and 50% (Bostock, 1986). Furthermore, the grading of mast cell tumours, particularly those of intermediate differentiation, is somewhat subjective, and there is marked discrepancy in the proportions of the various grades between series from different authors (Table I).

Nucleolar organiser regions are present in the nucleus of all cells, where they act as the sites for transcription of rRNA. They can be visualised readily in either impression smears or formalin fixed, routinely processed paraffin sections because of their close association with proteins containing a large number of disulphide bonds, which bind to silver ions (Smith *et al.*, 1988). They are, therefore, usually referred to as AgNORs when stained in this way.

Crocker and his colleagues have shown that the average number of AgNORs per nucleus correlates well with histological grading methods for a variety of human neoplasms, including lymphomas (Crocker & Nar, 1987), mammary tumours (Smith & Crocker, 1988) and melanomas (Crocker & Skilbeck, 1987) while others (Derenzini *et al.*, 1988) have similarly demonstrated an increased number in intestinal adenocarcinomas compared with hyperplastic polyps.

In view of these correlations we considered that the number of AgNORs might be of value as a prospective indicator of prognosis in canine mast cell tumours, particularly in view of their ease of demonstration and objectivity of measurement.

In dogs, most mast cell tumours which are destined to kill their host do so within 6 months of initial excision as a result either of inoperable local recurrence in the scar, or lymphatic metastasis (Bostock, 1973) so that an assessment of predictive value for AgNORs should be obtained much sooner in dogs than for comparable human neoplasms in which follow-up studies in excess of 5 years are normally required.

### Materials and methods

Spontaneously arising subcutaneous mast cell tumours were excised by veterinary surgeons in general practice, fixed in formal saline and submitted for histological examination. Data supplied at the time of submission included the breed, sex and age of the dog, the site and size of the tumour and a clinical assessment of tumour stage. Representative portions of each neoplasm were processed in the usual way and stained with haematoxylin and eosin. Following a diagnosis of mast cell tumour, 3 $\mu$m paraffin sections were prepared, stained with the silver colloid method previously described (Ploton *et al.*, 1986) and the number of AgNORs in 100 cells counted under oil immersion ($\times$1,000), in random nuclei. This technique of enumeration can now be regarded as standard, and is preferable to the use of image analysis systems which are often unable to distinguish between adjacent AgNOR dots (Crocker *et al.*, 1989*a*).

Tumours were sub-divided histologically into well differentiated, intermediate and poorly differentiated grades using the criteria described previously (Bostock, 1973) and the mitotic index was assessed by counting the number of cells in mitosis in 10 high power fields at $\times$400 magnification, selected at random. All tumours were graded, and mitotic indices assessed before the AgNOR count was known.

All dogs in clinical stage To No Mo (Owen, 1980) immediately after surgery were followed up at 8 week intervals by means of telephone interviews with the referring veterinary surgeon and owner, and the presence of recurrence, intercurrent disease or death noted. The cause of death was ascertained by the referring veterinarian. No dogs were allowed to die naturally and where death is referred to in the text or tables it indicates euthanasia in response to inoperable local recurrence or disseminated metastases.

### Results

Fifty dogs with histologically confirmed mast cell tumours which showed no clinical evidence of residual tumour immediately after surgery were followed up until death, or for a minimum of 9 months.

Nineteen tumours were well differentiated histologically, 16 were intermediate and 15 poorly differentiated, the mean

Correspondence: D.E. Bostock.
Received 21 October 1988, and in revised form, 3 February 1989.

**Table I** Distribution of tumour grades in histologically evaluated spontaneous mast cell tumours

| | | *Number of dogs (%) with* | | | |
|---|---|---|---|---|---|
| *Author* | *Year* | *Well differentiated* | *Intermediate* | *Poorly differentiated* | *Total* |
| Hottendorf & Neilsen | 1967 | 161 (54) | 82 (27) | 57 (19) | 300 |
| Bostock | 1973 | 39 (34) | 30 (26) | 45 (40) | 114 |
| Patnaik *et al.* | 1984 | 17 (20) | 36 (44) | 30 (36) | 83 |

AgNOR counts per cell in the three groups being 1.4, 3.2 and 6.3 respectively (Figures 1 and 2). The difference between each of these was significant at the $P=0.0001$ level (Student's $t$ test, Table II).

The AgNOR count varied from an average of 1.1 to 8.1 per cell, the relationship between the quintiles of these counts, median survival time and tumour related death rate being shown in Table III. From this it can be seen that the dogs fell into three distinct groups in terms of both tumour related deaths and median survival time. Animals with a count of 4.9 or above had a poor prognosis, with 3/4 dying as a direct result of the tumour within the first 4 months after surgery. Those with intermediate counts, between 1.7 and 4.8, had a better prognosis although about 1/3 were destroyed because of tumour related disease, while no dog with a count of less than 1.7 has been destroyed because of the tumour to date.

**Figure 1** Well differentiated mast cell tumour. Most nuclei contain 1–3 AgNORs (×700).

**Figure 2** Poorly differentiated mast cell tumour. Many nuclei contain multiple small AgNORs (×700).

When the animals were divided into high and low count groups, the former consisting of dogs with a count of 4 or more and the latter those with a count less than 4, there was a highly significant difference in both survival time and tumour related death rate (Table IV), so that for general prognostic purposes division into these two groups would be adequate.

The mitotic index of some canine tumours is known to be of prognostic significance. The survival time of dogs with mast cell tumours in relation to the mitotic index of the primary neoplasm is shown in Table V. The index varied from 0 to 64 but there was no significant difference between animals with tumours of mitotic index 0–4 or 5–10. However, tumour related death rates were significantly worse ($P<0.05$, $\chi^2$) for dogs with tumours of mitotic index greater than 10. Two-thirds of the dogs with lesions of high mitotic index were destroyed because of tumour related disease compared with 1/4 animals with low mitotic index tumours.

It is generally recognised that canine mast cell tumours have a marked breed predisposition, with boxers and Labrador retrievers being over-represented in most surveys, and that the prognosis for boxers is generally better than that for other breeds. This is, however, entirely due to the relatively high proportion of well differentiated tumours in boxers, and tumours of similar grade carry the same prognosis, regardless of breed (Bostock, 1973). This is further illustrated in Table VI, by the very close similarity in AgNOR count between tumours of the same grade, regardless of breed.

### Discussion

In man, it has generally been found that AgNORs are greater in number (and smaller in size) in high-grade than low-grade malignant neoplasms. In most species, NORs are restricted to a constant number of acrocentric chromosomes and it would be anticipated that, with increasing ploidy, AgNOR numbers would be elevated. However, it has been shown, by means of DNA flow cytometry, that there is no relation between NOR numbers and DNA ploidy in human lymphomas (Crocker *et al.*, 1988). Conversely, in the same study, a close correlation between NOR numbers and percentage S phase cells was found, suggesting that NOR numbers are related to cellular proliferation. A high positive correlation between AgNOR numbers and the number of cells labelled by the proliferation-marking monoclonal antibody, Ki67, has also been demonstrated (Hall *et al.*, 1988). Furthermore, it has recently been shown that individual human lymphoid cells with a high AgNOR count are also Ki 67 positive (Crocker *et al.*, 1989*c*).

In addition, lymphomas with high interphase NOR counts do not necessarily possess an excess of metaphase NORs on chromosomes, whilst specimens with low interphase AgNOR numbers often have hyperdiploid chromosomal complements and excessive metaphase AgNORs (Crocker *et al.*, 1989*b*), again illustrating the lack of correlation between interphase and metaphase or chromosomal NOR counts.

NOR numbers have also been shown to reflect cellular differentiation experimentally. When a human promyelocytic leukaemia cell line (HL60) was induced to differentiate to granulocyte-like cells by dimethylsulphoxide, the number of AgNORs diminished (Reeves *et al.*, 1984). While it is still uncertain whether an increase in interphase NOR number is fundamentally associated with neoplastic transformation, the

**Table II** Correlation between tumour grade, AgNOR count and survival time for dogs with spontaneous mast cell tumour

| *Histological grade* | *Number of dogs (%)* | *Mean AgNOR count (range)* | *Median survival time (weeks)* | *Number (%) dead from tumour* |
|---|---|---|---|---|
| Well differentiated | 19 (38) | 1.4 (1.1–3.5) | 40[a] | 2 (10) |
| Intermediate | 16 (32) | 3.2 (2.5–4.2) | 36[a] | 4 (25) |
| Poorly differentiated | 15 (30) | 6.3 (5.1–8.1) | 13 | 11 (73) |

[a]Median dog still alive at time of writing.

**Table III** Quintiles of AgNOR counts in relation to survival time and cause of death for dogs with spontaneous mast cell tumours

| *Count* | *No. of dogs* | *Median survival time (weeks)* | *Dead from tumour (%)* | *Significance of difference* ($\chi^2$) | *Still alive at time of writing* |
|---|---|---|---|---|---|
| 6.5–8.1 | 4 | 5 | 3 (75) | | 1 |
| 4.9–6.4 | 11 | 17 | 8 (72) | $P<0.05$ | 3 |
| 3.3–4.8 | 7 | 36[a] | 2 (35) | | 5 |
| 1.7–3.2 | 13 | 36[a] | 4 (32) | $P<0.05$ | 7 |
| <1.7 | 15 | 62[a] | 0 (0) | | 14 |
| Total | 50 | | 17 (34) | | 30[b] |

[a]Median dog still alive at time of writing; [b]Three dogs were destroyed because of unrelated disease, without evidence of tumour recurrence.

**Table IV** The relationship between AgNOR count, median survival time and tumour death rate in dogs with spontaneous mast cell tumours

| *Count* | *No. of dogs* | *Median survival time (weeks)* | | *Number dead from tumour (%)* | |
|---|---|---|---|---|---|
| ⩾4 | 18 | 17 | $P<0.01$ (rank sum test) | 12 (66) | $P=0.001$ ($\chi^2$ test) |
| <4 | 32 | 50[a] | | 5 (15) | |

[a]Median dog still alive at the time of writing.

**Table V** The relationship between survival time tumour related death rate and mitotic index for dogs with spontaneous mast cell tumours

| *Mitotic index* | *No. of dogs* | *Median survival time (weeks)* | *No. dead from tumour (%)* |
|---|---|---|---|
| 0–4 | 35 | 40[a] | 8 (23) |
| 5–10 | 4 | 40[a] | 1 (25) |
| >10 | 11 | 11 | 8 (72) |

[a]Median dog still alive at time of writing.

**Table VI** The relationship between breed of dog and AgNOR count for tumours of the same grade

| | | *Mean count for grade* | | |
|---|---|---|---|---|
| *Breed* | *No. of dogs* | *Well differentiated* | *Intermediate* | *Poorly differentiated* |
| Boxer | 10 | 1.5 | 3.0 | 6.7 |
| Labrador/retriever | 21 | 1.4 | 3.0 | 6.5 |
| Other pure breed | 10 | 1.3 | 3.8 | 6.4 |
| Cross bred | 9 | 1.7 | 3.1 | 5.8 |

strong correlation with both cellular differentiation and malignant behaviour demonstrated in this study suggests that this may be so.

Although these results again reveal three different groups of mast cell tumour which are closely correlated with tumour grade, the objectivity of the method should greatly enhance grading and enable those dogs which require prophylactic post-surgical therapy to be identified with more certainty.

A further advantage of the technique is its ready application to cytological preparations, which are extremely difficult to grade using conventional stains. The value of the method has been demonstrated in the differentiation of malignant cells from reactive in patients with pleural effusions (Ayres et al., 1988) and an evaluation of its use in needle aspirates from canine mast cell tumours is currently being undertaken.

The observation that mitotic index is of prognostic significance is also of interest, although this feature alone is a relatively insensitive measure of behaviour for tumours of lower mitotic index. Some lesions with high AgNOR counts had a low mitotic index and their behaviour was more accurately predicted by the AgNOR count than the mitotic index.

### References

AYRES, J.G., CROCKER, J. & SKILBECK, N. (1988). Evaluation of the Ag NOR technique in the diagnosis of malignant mesotheliomas. *Thorax*, **43**, 366.

BOSTOCK, D.E. (1973). The prognosis following removal of mastocytomas in dogs. *J. Small Anim. Pract.*, **14**, 161.

BOSTOCK, D.E. (1986). Neoplasms of the skin and subcutaneous tissues in dogs and cats. *Br. Vet. J.*, **142**, 1.

CROCKER, J., BOLDY, D.A.R. & AYRES, J.G. (1989*a*). How should we count AgNORs? Proposals for a standard approach. *J. Pathol.* (in the press).

CROCKER, J., JANMOHAMED, R.M.I., ARMSTRONG, S.J., HULTEN, M. & LEYLAND, M.J. (1989*b*). The relationship between numbers of interphase NORs and NOR-bearing chromosomes in non-Hodgkin's lymphoma. *J. Pathol.* (in the press).

CROCKER, J., McCARTNEY, J.C. & SMITH, P.J. (1988). Correlation between DNA flow cytometric and nucleolar organizer region data in non Hodgkins lymphomas. *J. Pathol.*, **154**, 151.

CROCKER, J., MURRAY, P.G. & BOLDY, D.A.R. (1989*c*). Sequential labelling with monoclonal antibodies (including Ki67) and demonstration of AgNORs in frozen sections. *J. Pathol.* (in the press).

CROCKER, J. & NAR, P. (1987). Nucleolar organizer regions in lymphomas. *J. Pathol.*, **151**, 111.

CROCKER, J. & SKILBECK, N. (1987). Nucleolar organizer region associated proteins in cutaneous melanotic lesions: a quantitative study. *J. Clin. Pathol.*, **40**, 885.

DERENZINI, M., ROMAGNOLI, T., MINGAZZINI, P. & MARINOZZI, V. (1988). Interphase nucleolar organizer region distribution as a parameter to differentiate benign from malignant epithelial tumours of human intestine. *Virchows Arch.* (*Cell Pathol.*), **54**, 334.

HALL, P.A., CROCKER, J., WATTS, A. & STANSFIELD, A.G. (1988). A comparison of nucleolar organizer region staining and Ki 67 immunostaining in non-Hodgkin's lymphoma. *Histopathology*, **12**, 373.

HOTTENDORF, G.H. & NEILSEN, S.W. (1967). Pathologic study of 300 extirpated canine mastocytomas. *Zentralbl. Veterinarmed.* (*A*), **14**, 272.

OWEN, L.N. (1980). *TNM Classification of Tumours in Domestic Animals*. World Health Organization: Geneva.

PATNAIK, A.K., EHLER, W.J. & MACEWEN, E.G. (1984). Canine cutaneous mast cell tumour: morphologic grading and survival time in 83 dogs. *Vet. Pathol.*, **21**, 469.

PLOTON, D., MENAGER, M., JEANNESSON, P., HIMBER, G., PIGEON, F. & ADNET, J.J. (1986). Improvement in the staining and in the argyrophilic protein of the nucleolar organizer region at the optical level. *Histochem. J.*, **18**, 5.

REEVES, B.R., CASEY, G., HONEYCOMBE, J.R. & SMITH, S. (1984). Correlation of differentiation state and silver staining of nucleolar organizers in the promyelocytic leukaemia cell line HL-60. *Cancergenet. Cytogenet.*, **13**, 159.

SMITH, P.J., SKILBECK, N., HARRISON, A. & CROCKER, J. (1988). The effect of a series of fixatives in the Ag NOR technique. *J. Pathol.*, **155**, 109.

SMITH, R. & CROCKER, J. (1988). Evaluation of nucleolar organizer region-associated proteins in breast malignancy. *Histopathology*, **12**, 113.